# Experiences and management strategies of Norwegian GPs during the COVID-19 pandemic: a longitudinal interview study

**DOI:** 10.1080/02813432.2022.2142796

**Published:** 2022-11-09

**Authors:** Silje Rebekka Heltveit-Olsen, Lene Lunde, Anja Maria Brænd, Ivan Spehar, Sigurd Høye, Ingmarie Skoglund, Pär-Daniel Sundvall, Guro Haugen Fossum, Jørund Straand, Mette Bech Risør

**Affiliations:** a The Antibiotic Centre for Primary Care, Department of General Practice, Institute of Health and Society, University of Oslo, Oslo, Norway; b Department of Nursing Science, Institute of Health and Society, Faculty of Medicine, University of Oslo, Oslo, Norway; c Department of General Practice, Institute of Health and Society, University of Oslo, Oslo, Norway; d Department of Health Management and Health Economics, Institute of Health and Society, Faculty of Medicine, University of Oslo, Oslo, Norway; e Institute of Psychology, Oslo New University College, Oslo, Norway; f General Practice/Family Medicine, School of Public Health and Community Medicine, Institute of Medicine, Sahlgrenska Academy, University of Gothenburg, Gothenburg, Sweden; g Research, Education, Development and Innovation, Primary Health Care, Region Västra Götaland, Sweden; h General Practice Research Unit (AFE), Department of General Practice, Institute of Health and Society, University of Oslo, Oslo, Norway; i The Research Unit for General Practice, Department of Public Health, University of Copenhagen, Copenhagen, Denmark; j The General Practice Research Unit, Department of Community Medicine, UiT The Arctic University of Norway, Tromsø, Norway

**Keywords:** COVID-19, general practice, organization and management strategies, practice management, qualitative research

## Abstract

**Objective:**

When the COVID-19 pandemic reached Norway, primary health care had to reorganize to ensure safe patient treatment and maintain infection control. General practitioners (GPs) are key health care providers in the municipalities. Our aim was to explore the experiences and management strategies of Norwegian GPs during the COVID-19 pandemic - over time, and in the context of a sudden organizational change.

**Design:**

Longitudinal qualitative interview study with two interview rounds. The first round of interviews was conducted from September–December 2020, the second round from January–April 2021. In the first interview round, we performed eight semi-structured interviews with GPs from eight municipalities in Norway. In the second round, five of the GPs were re-interviewed. Consecutive interviews were performed 2–4 months apart. To analyze the data, we used thematic analysis.

**Results:**

The COVID-19 pandemic required GPs to balance several concerns, such as continuity of care and their own professional efforts. Several GPs experienced challenges in the collaboration with the municipality and in relation to defining their own professional position. Guided by The Norwegian Association of General practitioners, The Norwegian College of General Practice and collegial support, they found viable solutions and ended up with a feeling of having adapted to a new normal.

**Conclusions:**

Although our study demonstrates that the GPs adapted to the changing conditions, the current municipal health care models are not ideal. There is a need for clarification of responsibilities between GPs and the municipality to facilitate a more coordinated future pandemic response.

**Key Points**
- Facing the COVID-19 pandemic, the primary health care service in Norway had to reorganize to ensure safe patient treatment and maintain infection control.
- Several GPs experienced challenges in collaboration with the municipalities.
- There is a need for clarification of responsibilities between GPs and the municipality.

## Introduction

The novel coronavirus outbreak was declared a public health emergency of international concern by the World Health Organization (WHO) on the 30th of January 2020 [1]. One and a half months later, what was once a distant epidemic had rapidly expanded into an extensive pandemic [1,2]. The Norwegian health care system did not have organizational blueprints ready for managing a pandemic of this magnitude, nor sufficient access to test medium and personal protective equipment (PPE) [3]. To handle the pressing situation, the government imposed the strongest restrictions ever given in time of peace upon Norwegian residents on the 12th of March 2020 [4,5]. Effective infection prevention and control in the municipalities was a crucial strategy to contain the spread of the disease and avoid that the number of hospital admissions surpassed the capacity of secondary health care. Experiences from previous epidemics indicated that most patients would be handled in primary health care [6]. General practitioners (GPs) are key providers of health care in the municipalities, and gatekeepers to secondary health care [7]. It was important to minimize the risk of spread of the virus to vulnerable patients at health care clinics as well as protecting the GPs from infection, quarantine, and isolation. A great proportion of the GPs isolated or quarantined would result in reduced access to primary care, increase the pressure on secondary health care and challenge the quality and continuity of patient care in the municipalities [8].

There are significant differences in size, geography, available resources, and organization of health care services between the 356 municipalities in Norway. It was up to each municipality to solve the internal pandemic puzzle to ensure sound infection control management and adequate access to health care services for its inhabitants. Like in other countries, a new level of care was created in the primary health care system, as different versions of respiratory clinics were established [9]. For the duration of this study, all patients with symptoms of respiratory tract infections were attended at respiratory/COVID clinics in Norway as a part of a strategy to keep the infection out of the GP offices and out-of-hours clinics. In some municipalities, these clinics were co-located (though physically separated) in or in proximity to the out-of-hours clinic. As the nature of the COVID-19 virus was previously unknown, new knowledge emerged at a high pace. Based on short-lived medical truths, rules and regulations, GPs had to reorganize their practices and adapt their approach to patient care according to current infection prevention considerations [10,11]. Most striking was the explosive increase in the use of digital communication and remote consultations [12–14].

Primary health care services are mainly publicly funded in Norway. The municipalities have the responsibility for providing and organizing all primary health care services – including access to a GP [15]. Most GPs are self-employed with obligations to the municipality to provide health care services through the Regular General Practitioner Scheme (GP Scheme) [16,17]. The self-employed GPs manage their practices as autonomic businesses, often in groups of 3–5 GPs. Even though self-employment is the most usual form of organization, an increasing proportion of Norwegian GPs are employed by the municipality. Initially deemed a success by the patients after the introduction in 2001 [18], the GP Scheme have been under pressure in the resent years. Increasing work load and declining recruitment was already a challenge before the pandemic [19].

The rapid changes of the COVID-19 pandemic provoked sudden changes in overall organization in the municipalities, between the municipalities and the GPs, within the GP offices and between the GPs and their patients. In this study, the aim was to explore the experiences and management strategies of Norwegian GPs during the COVID-19 pandemic - over time, and in the context of a sudden organizational change.

## Materials and methods

### Study design

We performed a longitudinal qualitative interview study and developed a thematic, semi-structured interview guide for two interview rounds [20]. The first round of interviews was conducted from September to December 2020, the second round from January to April 2021. Subsequent interviews were performed 2–4 months apart.

### Research team

The research team was composed of five academic and clinical GPs (SHO, SH, AB, PS, IMS), an academic and clinical nurse (LL), an organizational researcher (IS), an academic and clinical otorhinolaryngologist (GH), a professor in general practice (JS), and a professor in medical anthropology (MR).

### Setting, participants and recruitment

We wanted to interview GPs in municipalities of different population size, geographic placement, and local spread of COVID-19. We divided all municipalities in Norway into three regions: north, middle, and south. Within each region, we grouped the municipalities according to size and spread of COVID-19 at the time of sampling. One GP from each of the groups in the three regions was invited by email and/or telephone through a process of a mixed purpose/convenience sampling, mainly through the municipality chief medical officer (MCMO). No GP abstained from participation when approached. We initially aimed for ten GP interviews in the first interview round. After eight interviews, the data collection was stopped as no new major themes were found during preliminary analytical discussions. Five of the GPs were re-interviewed in the follow-up round. This number of interviews was a pragmatic choice based on feasibility, time and resources available. Written informed consent from the GPs was obtained. Participant characteristics are displayed in Table 1.

**Table 1.** Characteristics of participating general practitioners (GPs).

Participant characteristics (total *n* = 8)	Sex	Age (years)	Experience	Number of patients on GP list	Number of GPs in the office	Details
Interview 1 (*n* = 8)	4 Female, 4 Male	31–56 (median 44)	≤5 years (*n* = 1) 6–20 years (*n* = 6) ≥21 years (*n* = 1)	602–1400 (median 1000)	3–8 (median 6)	8 General practitioners 5 Work in out-of-hours clinic^a^ 5 Work in COVID clinic^a^ 6 Work in municipal position^a^
Interview 2 (*n* = 5)	2 Female, 3 male	31–56 (median 44)	≤5 years (*n* = 1) 6–20 years (*n* = 3) ≥21 years (*n* = 1)	950–1400 (median 1050)	3–8 (median 6)	5 General practitioners 3 Work in out-of-hours clinic^a^ 3 Work in COVID clinic^a^ 4 Work in municipal position^a^

^a^Some GPs worked in out-of-hours clinics, respiratory/COVID clinics and/or part time in a municipal position in addition to their GP practice during the pandemic. GPs working part time as municipal chief medical officers (MCMO) at baseline were interviewed as MCMOs in the CovidNor study and are not included in this analysis.

### Data collection and analysis

We created an interview guide based on our aim, available knowledge of the COVID-19 pandemic, and our own clinical experience. The interview guide was sent to reference GPs for feedback and revised accordingly. One pilot interview was performed and discussed within the research group. This resulted in only minor revisions of the guide, and the interview was included in the study. The interviews were performed *via* Zoom [21], audio-taped digitally and transferred directly to a secure storage platform [22,23]. SHO and MR conducted the interviews. After each interview, a written summary was shared within the research team. After the first round of interviews, the research group discussed their experiences and planned the interview guide for the follow-up interviews. The second guide contained the same main themes and questions but was personalized for each participant to follow up on themes from the first interview. Two research assistants transcribed the interviews verbatim, and SHO re-listened to all audiotapes while proofreading the transcripts.

The data material was analyzed using Braun & Clarke thematic analysis [24], a qualitative method for identifying, analyzing, and reporting patterns (themes) within data. SHO, LL, SH, AB and MR read the transcripts searching for meanings and patterns. The first impressions of the material were discussed in a joint meeting. SHO and LL then did the initial coding using NVIVO software. The codes were discussed among SHO, LL, SH, AB and MR, and codes that fit together in preliminary themes were identified. The themes and codes were then reviewed and revised several times within the research team, before defining and naming definite themes. During this process, the transcripts were re-read by SHO and LL to validate the themes. We performed an inductive thematic analysis to look at patterns, meanings and implications across the data set using a semantic and realist approach [25]. The analysis was theorized by interpreting the significance and contextual framing of the themes. The study was reported in accordance with Standards for Reporting Qualitative Research (SRQR) [26].

#### Ethics

The study was presented for the Norwegian Ethical Committee (REK southeast C, ref; 155344) that found the project to be outside the scope of the Health Research Act, not requiring pre-approval. Data management and safety was approved by the Norwegian Centre for Research Data (NSD, ref; 615396).

## Results

This result section is divided into three themes representing different phases of the pandemic response from the initial shock through reorganization and adaption to a new normal. Each theme describes experiences, management strategies, process and change. The first two themes mainly represent reflections and analysis of the period from start of the pandemic to the first interview and the last theme represents the reflections made by the GPs in their follow-up interviews.

### Balancing precaution, prevention and continuity

When the pandemic approached Norway, an early reaction reported by the GPs was thinking the situation would unfold like previous viral pandemics. However, media reports from other European countries painted an alarming picture of congested intensive care units, rising death tolls and exhausted health care personnel.

It was a bit like Armageddon, just waiting for the meteor to land and impact. I think we were a bit in a state of emergency […] There was a lot of uncertainty. (GP02)

As knowledge about the disease and its potential consequences increased, the GPs expressed a shift towards concern about the local preparedness and ability to handle the pandemic. The GPs described a total collapse of the health care system as their ultimate fear, as overburdened health care services could potentially position health care personnel in ethically demanding situations. With the urgent situation in Europe as backdrop, the GPs described the feeling of waiting for a predicted disaster. Some GPs said it came like a shock when the pandemic started in Norway, and that the initial phases of the pandemic felt uncertain and chaotic. Following the lockdown on 12^th^ of March 2020, the flow of patients to the GP offices subsided. The nearly empty waiting rooms gave the GPs time to reorganize and adjust to the new situation. Patients disappearing from the GP offices also gave increasing cause for concern, as prolonged regulations keeping the patients away from their GP could yield negative health consequences. The GPs had to balance their clinical practice to keep up the infection prevention, but at the same time maintain adequate health care services.

The biggest challenge is really to make sure that people are not infected when they are in the doctor’s office or, the balancing act between being hysterical and being pragmatic. (GP02)

To fulfil their tasks, the GPs had to balance their own concerns, the fear for their employees and the patients to normalize the situation both emotionally and pragmatically. A coping-strategy for several GPs was to suppress thoughts of being infected and spreading the disease, while keeping adequate infection prevention measures. In practical work, a compromise was to accept patient assessments that were below normal medical standard, but good enough given the circumstances. Early in the pandemic, the GPs were worried for suboptimal follow-up of chronically ill patients and delay in diagnosis and treatment of new-onset disease.

Seeing things deteriorate that otherwise could have been kept fairly well in check with early intervention. It has been frustrating to watch. (GP03)

Weighing infection protection against the need for closer follow-ups and early intervention was challenging for the GPs. The GPs questioned where to set the bar for preventive measures and how long society could maintain strong restrictions without doing bigger damage to the health of the population than the virus itself. This resulted in conflicting emotions between the wish for precaution and the wish for continuity of care. While the GPs strived for normalization of the situation, they also revealed concerns about whether their efforts were sufficient. The GPs considered the pre-existing municipal pandemic plans outdated and deficient for management of the COVID-19 pandemic. Initial work on improvement of the plans was focused on a worst-case scenario. Despite the initial state of emergency and fear of collapse, the GPs pointed out that few of the dismal predictions became a reality in the Norwegian health care system. The GPs experienced that this worst-case strategy left the municipalities without clear guidelines for management of the early pandemic outbreaks where many patients were isolated, and/or quarantined presenting milder symptoms, but few in need of hospitalization.

### Involved or a fly in the ointment?

Overall, the pandemic represented a continuous challenge that put pressure on GPs’ work environment, collaboration, and patient management, and brought forward several management challenges at different levels. The GPs had to reorganize their practices almost overnight, most notably by swift digitalization of communication and measures to ensure infection prevention and control in the GP offices. Several self-employed GPs experienced that their dual relationship with the municipalities gave rise to unclear responsibilities in the collaboration process in the early phase of the pandemic. This both applied to access to PPE, information flow, organization of the GP offices and staffing of respiratory/COVID clinics. In some municipalities, the GPs experienced that the collaboration went in a near seamless manner, whereas other GPs experienced great challenges in terms of role definition and responsibilities in the pandemic response. In the initial phases of the pandemic, the information from the National Institute of Public Health (NIPH), the government and the media were extensive. Most GPs found it difficult to navigate the guidelines to find specific advice for general practice. The GPs described how they expected the municipality to provide guidance for the GPs as part of a coordinated municipal pandemic response. However, several GP experienced that the information from the municipality was limited and that the role of the Municipality Chief Medical Officer (MCMO) was not scaled for the pandemic. Some GPs described how the municipality quickly took action to correct this, whereas others experienced lengthy periods of one-way communication and a feeling of being left alone to figure out what to do. Some GPs also expressed that it could be challenging to know who to contact within the municipal organization, and that the response on similar requests could differ depending on who you got a hold on. In this situation, The Norwegian Association of General Practitioners (GP Association) and The Norwegian College of General Practice (GP College) took action to fill in the information void.

I think the general practitioners’ association has been outstanding. They have given me the information I really need. And what our role is. Using public health [NIPH] does not completely give me the specific information that I need. (GP05)

All the GPs emphasized the importance of the information and guidelines originating from the GP Association and GP College. The information and guidelines were adapted to a clinical setting by peers with first-hand knowledge of general practice, and the GPs felt that it could better accommodate their needs. The pandemic forced the GPs to reorganize their practices to protect themselves and their patients from infection. It was important for the GPs to be coordinated when establishing new routines.

We are all different so the biggest challenge maybe, is to get all to agree and do the same. (GP06)

A recurring theme in the interviews was that the rapid changing situation enhanced the daily interaction between and within GP offices. When specific advice was unavailable or the guidelines were unclear, the GPs collaborated and supported each other. Knowing that the GPs within a municipality had comparable routines was reassuring in an otherwise unpredictable situation. Formal and informal meetings were important for the GPs to reach consensus on the practical interpretation of the current guidelines and plan the day or week ahead. To be coordinated was important not only for the work environment, but also for the patients. A large difference in practice between GPs in the same municipality would increase uncertainty in the population.

The establishment of respiratory/COVID clinics represented a new level of care in the municipalities. To be split between claiming value for work and to do unpaid voluntary work put some GPs in an ethical dilemma positioning them as demanding for wanting clear agreements to regulate this extra work:

We get the impression that there are many in the municipality who think that we are, in a way, demanding, a fly in the ointment, for wanting to get paid to do a job. And want compensation for that what we do is risky and that we do not have the same rights as employees. But no one understands that. (GP04)

Some GPs expressed ambivalence about making demands to compensate for increased risk and workload amid an ongoing crisis and thus be perceived as pandemic profiteers. These conflicting emotions originated from different mutual expectations between the GPs and the municipalities. How the municipalities involved the GPs in the pandemic response affected the GPs’ views of the collaboration with the municipality and the success of the response. Although perceived as challenging, the GPs preferred to be involved rather than standing on the sideline. Where the GPs were left with a sense of poor collaboration with the municipality, the GPs reported a lack of involvement in the municipal pandemic response as a contributing factor to dissatisfaction. These municipalities fulfilled their obligations regarding infection prevention and control for its inhabitants without involving the GPs in the process.

The recognition of the GPs role as part of the municipal health service is absent. And when the crisis came the municipality started to deal with the COVID pandemic all alone. And with no conscious thought about what role the GP should have. (GP07)

Other GPs described the dual relationship between the self-employed GPs and the municipality as feeling a bit on the side of the municipality. The lack of involvement of the GPs was also perceived as a lack of acknowledgement of their importance in the pandemic response, and that their input was not appreciated. This left the GPs with a sense of being treated unfairly; to be set aside, but still expected to just fix things on behalf of the municipality. Some GPs also pointed out the underused potential of the GPs to contribute to planning and coordinating the pandemic response. In municipalities where the GPs experienced good collaboration, the GPs described a feeling of unity, positive spirit, and fellowship within the municipality.

When something like that happens, when you see that an insane amount of talented people pull in the same direction as yourself. […] There was such good atmosphere and fellowship. We had to make it happen. (GP08)

These GPs described a proactive municipality initiating dialogue through regular meetings and dissemination of relevant information from the municipal activities to the GPs. In this way, the municipality took responsibility to maintain adequate GP services by actively involving the GPs in the municipal pandemic response. Consequently, the GPs described fewer challenging situations, less frustration, a closer collaboration with the MCMO and greater satisfaction with the local pandemic management. The difficulties in collaboration between the GPs and the municipality were described as general and pre-existing challenges enhanced by the pandemic. In the follow up interviews, the GPs suggested that structural factors in the organization of the municipality might have hampered a more coordinated interaction and collaboration in primary health care. Their proposal to overcome an obsolete and cumbersome municipality system was to bring in people with knowledge of the GP Scheme to assist the management and coordination of the response.

### Making order out of chaos – adapting to a new normal

The unpredictability of the pandemic was challenging for the GPs, and temporary knowledge and volatile regulations and guidelines provided ground for uncertainty. However, several GPs pointed out that adjusting to different situations and managing risks and uncertainty had always been a part of their work.

I think that, when you are a GP you can handle it better than many others, because we are quite used to handling many different issues, where you may not find any textbook answers. You have to be able to improvise and endure stress and pressure over time. So, that did not throw me off my game, I think. (GP08)

The act of improvising and finding solutions without a clear-cut answer was described as embedded in the role of the GP alongside the ability to handle stressful situations. Several GPs experienced that they, on a personal level, were quite comfortable with managing changing conditions. The GPs expressed that the versatility in the GP role and the organization in the GP Scheme enabled them to restructure and prioritize to adapt to variable conditions. During the first year of the pandemic, the GPs underwent a process from crisis through reorganization and adaptation to a new normality. In the follow up interviews, the GPs expressed a stronger feeling of preparedness.

We are getting used to living in a different situation. As long as we have some ideas about how to do things when it changes, that we have a plan to back us up. Then we cope really well with a little unpredictability. (GP04)

The enhanced feeling of preparedness was linked to having pandemic plans and structural organization in place. If these presuppositions were met, the GPs adapted well to change and unpredictability. The initial concern for suboptimal treatment of patients and lack of continuity had subsided in the follow-up interviews, as the GPs experienced a greater overview of their patient list, normalization of patient care and more control of the situation. The GPs also emphasized that vaccination of health personnel had an impact on the normalization process reducing both their own concerns as well as the concerns of their patients. The lack of specific information targeting the GPs was perceived less challenging as the GPs were getting used to changing information. The amount of information available was still perceived as large, and the GPs expressed limited capacity to follow up on the information flow. A coping mechanism was to choose the sources of information more carefully than in the early phases of the pandemic. The GPs who initially experienced poor collaboration with the municipality did not report an increased involvement in the pandemic response at time of the follow up interviews. However, the GPs now described the feeling of having enough basic knowledge to handle the pandemic, and that improving relations between the GPs and the municipality was an ongoing and general process not just concerning the management of the COVID pandemic. The main focus in the first phases of the pandemic was to manage the urgent situation. When approaching a new normality, pre-existing challenges came back up to the surface.

I have always worked a lot and been engaged in several ways. What I am experiencing now is that the bucket is full in another way. […] It is noticeable that there are more [GPs] who are struggling. (GP07)

Several GPs described a GP Scheme that was under pressure even before the pandemic. The call for reinforcement of the GP Scheme had been voiced for years, and the GPs experienced the pandemic as an additional load on the system. The GPs pointed out that the challenges had been allowed to build up over time. Increasing workload, lack of staff and difficulties with recruitment was already a problem before the pandemic. Although the GPs managed to create innovative solutions and adapt to a new normal, the pre-existing challenges were enhanced and made more visible by the pandemic. The workload had always been large, but now it was described as overpowering. Despite these struggles, the GPs felt they were able to provide adequate patient care throughout the pandemic.

## Discussion

### Principal findings

The COVID-19 pandemic, affecting both GPs and their patients, required GPs to balance several concerns, such as continuity of care and their own professional efforts of coping with the situation.

Several GPs experienced challenges of collaboration with municipalities and defining their own professional position, i.e. being more than ‘a fly in the ointment’. Guided by their GP Association, GP College and own practice support, they found viable solutions.

During the pandemic, GPs improvised and handled several stressful situations, ending up with a feeling of having adapted to a new normal.

## Strengths and limitations

We consider the longitudinal interview format to be a strength in this study. The design gave us the opportunity to follow up on experiences and management strategies over time. As a part of the design, the interview guide was revised prior to the follow-up interviews to reflect the preliminary analysis of the first round and focus on issues we wished to elaborate as longitudinal aspects. Several considerations of when to interview and who to recruit were done as well as part of the design. During analysis we ended up with a mainly cross-sectional description and a narrative thematic approach, however, we also were able to show how the GPs based their ‘new normal’ on mechanisms such as adaption, coping, reorganization and evaluation of their roles. Even though we did not integrate theory from the start as recommended [27], we believe we moved beyond the data during our discussions on management and roles.

The study was carried out in times of strict infection prevention measures making travel and physical meetings impossible. The use of digital platforms might be perceived as a weakness as it may have impacted the interaction with the GPs potentially masking subtle nonverbal communication. The personal interaction was also sometimes disturbed by brief technical difficulties. On the other hand, the use of digital communication might also have lowered the bar for what the GPs were willing to share, and it offered them an easy logistical solution for participation.

The first author and several of the co-authors are GPs. This can be considered a strength as the authors have in-depth knowledge of the GP Scheme and municipal organization. However, the study process, from planning to analysis, are indisputably influenced by our preconceptions as GPs. The in-depth knowledge about GPs and the Norwegian health system helped direct the research questions and the interview guide for the study. This may also have narrowed the focus and made us blind to some issues despite rounds of critical review of the interview guide in the research group. Doing the interviews, it was an advantage to be an insider to know what to follow up on, while trying all the time to stay open for topics that were outside the guide. A few interviews were done by MR, who is not a GP, but these interviews were quite similar to the rest. We believe that the interdisciplinary composition of the research group added to the reflexivity during the analytic process. During analysis we discussed coding and themes in several meetings to decide on the final themes. Although our preconceptions may have enhanced the wish to come up with discussions on how to solve problematic issues and what role the GPs play in this, we had focus on trying to be very open-minded to surprises in the data material. We also constantly discussed how to avoid ‘GP heroism’, balanced with tendencies in the data of making political statements or positioning GPs as underdogs. We tried to focus on the general patterns in line with our research questions, and in relation to the whole context of the pandemic.

### Findings in relation to theory and other studies

In the literature, we find several similarities of how GPs managed and responded to the pandemic [28]. The unfamiliarity with the corona virus created uncertainty and increased stress for GPs [10,29–31]. A review focusing on evidence from previous epidemics demonstrated a similar trend suggesting this reaction is not unique for the current pandemic [32]. An initial emotional response of crisis and state of emergency reported by the GPs in our study is thoroughly described also by Davies et al. [33]. As strong restrictions and lockdown were introduced, reducing the personal threat, the GPs in our study also expressed ambivalence and increasing concern for continuity of care for their patients. Indeed, several studies have reported a reduction in general practice consultations following periods of lockdown, partly counteracted with a swift digital reorganization [12–14,34,35]. In the initial wave in Germany, Linde et al. explored the diversity of opinions of GPs on the threat and measures against COVID-19. Linde’s study divides the GPs attitudes into four archetypes with ‘balancers’ being the predominant type (54%). These GPs rated the threat high, but were also more critical towards the potential consequences of strict preventive measures [36]. This is in accordance with our findings where the GPs emphasized the need for balancing out their own concerns pragmatically and normalize the situation to be able to provide adequate care for their patients. However, the GPs in our study reported that physical attendance leveled out through the course of the pandemic and that they felt that patients mostly received their usual care. A similar trend, with initial reduction of consultations challenging the continuity followed by a rather quick normalization, is also demonstrated in other countries [37]. A recurring theme in GP studies is the perceived lack of guidance and coordination from central authorities somewhat neglecting the primary health care sector [38–40]. When clear and suitable guidelines for practice did not come from the municipality and/or NIPH, the GPs in our study turned to the GP Association, GP College and their colleagues and enhanced the collaboration within and between GP offices. Comparable pragmatical solutions were also demonstrated in other countries [33,41,42].

Organizational change often requires participants to take on new tasks and roles. This was also the case for the GPs in our study. Organizational researcher Henry Mintzberg has studied the roles and behaviors that managers often engage in, and has classified these roles into three categories, namely interpersonal, informational and decisional roles [43].

Although not formally employed as managers, GPs exercise and combine several of these roles. According to Yukl, physicians manage on a daily basis, for example through planning, coordinating tasks and problem solving [44]. Mintzberg also notes that physicians are involved in decision-making that places them ‘squarely in the realm of management’. Therefore, Mintzberg’s role framework is helpful to interpret our findings [45].

During the pandemic, we found that the GPs had to keep up with rapid changing information and translate theory into clinical practice. According to the role framework, they utilized their informational role to understand and make use of the information. However, they also had to fill decisional roles to handle change through innovations and alternative solutions. Traditionally, GPs have a high degree of autonomy, and they have sought to acquire and retain power and autonomy over their profession [46,47]. This autonomy seems to be an advantage for GPs when exercising the entrepreneurial role to manage uncertainty and find alternative and innovative solutions to handle the ongoing pandemic. There are tensions between the GPs’ wish for autonomy and wish for clearer lines of responsibility within the municipal organization. If GPs are to have clearer lines of responsibility, this entails reduced autonomy (e.g. by GPs being employed by the municipality instead of being self-employed, or by being formally subordinated to the MCMO). At the same time, it seems that the current autonomy in the GPs role made the GPs better suited to face the pandemic, as also noted by Wanat et al. [41].

In a recent study exploring conditions for MCMO involvement in quality improvement in general practice, our research group found that MCMOs also call for more specific guidelines in relation to their own role vis-à-vis the municipality administration and the GPs [48]. The MCMOs in that study identified themselves as advisors and intermediaries between the municipality and the GPs. However, the MCMOs also emphasized their respect for GP autonomy and did not express a wish for more professional supervision or management responsibilities over the GPs. The question is then how clearer lines of responsibility in the municipal health service can be established. This is especially important in emergency situations where immediate action is needed. Several GPs in our study emphasized the wish for advisors with in-depth knowledge of the GP Scheme in the municipal organization to assist in the coordination between the municipalities and the GPs. Revisiting Mintzberg’s role typology, the GPs point out the need for someone (other than the MCMOs) to act as liaisons. This role does not seem to be adequately filled in the current municipal organization. The need for such liaisons is probably greater in larger municipalities, whereas in smaller municipalities, the chain of command is shorter making the organization better suited for involvement of the GPs through dialogue and close collaboration.

### Implications for practice, policies and research

To be better prepared for future pandemics, there is a need to clarify roles and responsibilities in the municipal organization. The GPs may represent an underused potential in coordination and management of future pandemics. However, the GPs’ preference for increased involvement must be balanced against their determination to maintain autonomy.

## Conclusions

Although our study demonstrates that the GPs adapted to the changing conditions through collaboration with colleagues and their own GP Association and GP College, the current municipal health care organization models are not ideal. Future discussions need to include responsibility clarification between GPs and the municipality to facilitate a more coordinated pandemic response.

## References

[1] WHO. Timeline: WHO's COVID-19 response 20. Available from: https://www.who.int/emergencies/diseases/novel-coronavirus-2019/interactive-timeline#event-43.

[2] NRK. First case of covid infection in Norway ("Første tilfelle av coronasmitte i Norge") 2020. Available from: https://www.nrk.no/norge/forste-tilfelle-av-koronasmitte-i-norge-1.14920058.

[3] Alsnes IV, Munkvik M, Flanders WD, et al. How well did Norwegian general practice prepare to address the COVID-19 pandemic? Fam Med Com Health. 2020;8(4):e000512. 10.1136/fmch-2020-000512 PMC7722348 33277356

[4] The Norwegian Government. Comprehensive measures to combat the coronavirus ("Omfattende tiltak for å bekjempe koronaviruset") [cited 2020 Oct 06]. Available from: https://www.regjeringen.no/no/aktuelt/nye-tiltak/id2693327/.

[5] Ursin G, Skjesol I, Tritter J. The COVID-19 pandemic in Norway: the dominance of social implications in framing the policy response. Health Policy Technol. 2020; 2020/12/01/9(4):663–672. 32874857 10.1016/j.hlpt.2020.08.004 PMC7452841

[6] Simonsen KA, Hunskaar S, Sandvik H, et al. Capacity and adaptations of general practice during an influenza pandemic. PLoS One. 2013;8(7):e69408. 23874960 10.1371/journal.pone.0069408 PMC3715475

[7] Sripa P, Hayhoe B, Garg P, et al. Impact of GP gatekeeping on quality of care, and health outcomes, use, and expenditure: a systematic review. Br J Gen Pract. 2019;69(682):e294–e303. 30910875 10.3399/bjgp19X702209 PMC6478478

[8] Matenge S, Sturgiss E, Desborough J, et al. Ensuring the continuation of routine primary care during the COVID-19 pandemic: a review of the international literature. Fam Pract. 2021;39(4):747–761. 10.1093/fampra/cmab115 PMC8515263 34611708

[9] Hibberd J, Carter J, McCoy M, et al. General practice in the time of COVID-19: a mixed-methods service evaluation of a primary care COVID-19 service. IJERPH. 2021;18(6):2895. 33809000 10.3390/ijerph18062895 PMC7998968

[10] Renaa T, Brekke M. Restructuring in a GP practice during the COVID-19 pandemic – a focus-group study. Tidsskr Nor Laegeforen 2021;141(2). 10.4045/tidsskr.20.0713 33528128

[11] Tsopra R, Frappe P, Streit S, et al. Reorganisation of GP surgeries during the COVID-19 outbreak: analysis of guidelines from 15 countries. BMC Fam Pract. 2021;22(1):96. 34000985 10.1186/s12875-021-01413-z PMC8127252

[12] Saint-Lary O, Gautier S, Le Breton J, et al. How GPs adapted their practices and organisations at the beginning of COVID-19 outbreak: a french national observational survey. BMJ Open. 2020;10(12):e042119. 10.1136/bmjopen-2020-042119 PMC7712933 33268433

[13] Alexander GC, Tajanlangit M, Heyward J, et al. Use and content of primary care office-based vs telemedicine care visits during the COVID-19 pandemic in the US. JAMA Netw Open. 2020;3(10):e2021476-e2021476. 33006622 10.1001/jamanetworkopen.2020.21476 PMC7532385

[14] Sigurdsson EL, Blondal AB, Jonsson JS, et al. How primary healthcare in Iceland swiftly changed its strategy in response to the COVID-19 pandemic. BMJ Open. 2020;10(12):e043151-e043151. 10.1136/bmjopen-2020-043151 PMC7722808 33293329

[15] HOD. Act on municipal health and care services ("Lov om kommunale helse- og omsorgstjenester"). 2011. Available from: https://lovdata.no/dokument/NL/lov/2011-06-24-30.

[16] Sandvik H. The Norwegian regular general practitioner scheme. Fam Med Primary Care Rev. 2006;8:314–319.

[17] HOD. Regulations for The Regular General Practitiones Scheme in the municipalities ("Forskrift om fastlegeordning i kommunene"). 2012. Available from: https://lovdata.no/dokument/SF/forskrift/2012-08-29-842.

[18] Sandvik H. Evaluation of the GP reform 2001-2005: summary and analysis of the evaluation’s sub-projects ("Evaluering av fastlegereformen 2001-2005: sammenfatning og analyse av evalueringens delprosjekter"). Oslo; 2006. (Council TNReditor.)

[19] Lilleås E. The GP scheme: CPR minus ("Fastlegeordningen: HLR minus"). Tidsskr Nor Legeforen 2018; 138.

[20] Hermanowicz JC. The longitudinal qualitative interview. Qual Sociol. 2013; 2013/06/0136(2):189–208.

[21] Grøndahl E. Routines for use of Zoom for red data at the Univeristy of Oslo ("Rutiner for bruk av Zoom for røde data ved Universitetet i Oslo") [updated 2020 Jun 06; cited 2020 Nov 06]. Available from: https://www.uio.no/tjenester/it/sikkerhet/lsis/tillegg/rutiner/bruk-av-zoom-for-rode-data.html.

[22] UIO. Audio-tape with red data ("Ta opp lyd med røde data") [updated 2020 Feb 07; cited 2020 Nov 06]. Available from: https://www.uio.no/tjenester/it/adm-app/nettskjema/hjelp/tips-triks/fortroligedata-diktafon.html.

[23] UIO. Services for sensitive data (TSD) ("Tjenester for sensitive data") [cited 2020 Nov 06]. Available from: https://www.uio.no/tjenester/it/forskning/sensitiv/.

[24] Braun V, Clarke V. Thematic analysis. APA handbook of research methods in psychology. Vol 2: research designs: quantitative, qualitative, neuropsychological, and biological. APA handbooks in psychology®. Washington (DC): american Psychological Association; 2012. p. 57–71.

[25] Braun V, Clarke V. Using thematic analysis in psychology. Qualitative Research in Psychology. 2006;3(2):77–101.

[26] O’Brien BC, Harris IB, Beckman TJ, et al. Standards for reporting qualitative research: a synthesis of recommendations. Academic Medicine. 2014;89(9):1245–1251. 24979285 10.1097/ACM.0000000000000388

[27] Calman L, Brunton L, Molassiotis A. Developing longitudinal qualitative designs: lessons learned and recommendations for health services research. BMC Med Res Methodol. 2013; 2013/02/0613(1):14. 23388075 10.1186/1471-2288-13-14 PMC3598728

[28] Borek AJ, Pilbeam C, Mableson H, et al. Experiences and concerns of health workers throughout the first year of the COVID-19 pandemic in the UK: a longitudinal qualitative interview study. PLoS One. 2022;17(3):e0264906. 35294450 10.1371/journal.pone.0264906 PMC8926177

[29] Fernemark H, Skagerström J, Seing I, et al. Working conditions in primary healthcare during the COVID-19 pandemic: an interview study with physicians in Sweden. BMJ Open. 2022;12(2):e055035. 10.1136/bmjopen-2021-055035 PMC8829841 35135771

[30] Coenen L, Poel LV, Schoenmakers B, et al. The impact of COVID-19 on the well-being, education and clinical practice of general practice trainees and trainers: a national cross-sectional study. BMC Med Educ. 2022;22(1):108–108. 35183171 10.1186/s12909-022-03174-4 PMC8857395

[31] Sotomayor-Castillo C, Nahidi S, Li C, et al. General practitioners’ knowledge, preparedness, and experiences of managing COVID-19 in Australia. Infect Dis Health. 2021;26(3):166–172. 33676878 10.1016/j.idh.2021.01.004 PMC7891055

[32] Desborough J, Dykgraaf SH, Phillips C, et al. Lessons for the global primary care response to COVID-19: a rapid review of evidence from past epidemics. Fam Pract. 2021;38(6):811–825. 33586769 10.1093/fampra/cmaa142 PMC7928916

[33] Davies M, Carr D, Dugan J, et al. Support FOR general practitoners DURING covid-19. Ulster Med J. 2021;90(3):151–156. 34815593 PMC8581688

[34] Albert SL, Paul MM, Nguyen AM, et al. A qualitative study of high-performing primary care practices during the COVID-19 pandemic. BMC Fam Pract. 2021;22(1):237. 34823495 10.1186/s12875-021-01589-4 PMC8614080

[35] Hoerold M, Gottschalk M, Debbeler CM, et al. Healthcare professionals’ perceptions of impacts of the covid-19-pandemic on outpatient care in rural areas: a qualitative study. BMC Health Serv Res. 2021;21(1):1298. 34856970 10.1186/s12913-021-07261-y PMC8638652

[36] Linde K, Bergmaier C, Torge M, et al. The diversity of opinion among general practitioners regarding the threat and measures against COVID-19 – cross-sectional survey. Eur J Gen Pract. 2021;27(1):176–183. 34319199 10.1080/13814788.2021.1954155 PMC8330783

[37] Deml MJ, Minnema J, Dubois J, et al. The impact of the COVID-19 pandemic on the continuity of care for at-risk patients in swiss primary care settings: a mixed-methods study. Soc Sci Med. 2022;298:114858–114858. 35247784 10.1016/j.socscimed.2022.114858 PMC8868005

[38] Siebenhofer A, Huter S, Avian A, et al. COVI-Prim survey: challenges for Austrian and german general practitioners during initial phase of COVID-19. PLoS One. 2021;16(6):e0251736. 34111120 10.1371/journal.pone.0251736 PMC8191874

[39] Pilbeam C, Edwards G, Tonkin-Crine S, et al. Primary care preparedness for the SARS-CoV-2 pandemic: a survey of NHS GPs. Fam Pract. 2021;39(3):332–339. 10.1093/fampra/cmab145 PMC8828273 34871397

[40] Kurotschka PK, Serafini A, Demontis M, et al. General practitioners’ experiences during the first phase of the COVID-19 pandemic in Italy: a critical incident technique study. Front Public Health. 2021;9:623904–623904. 33614587 10.3389/fpubh.2021.623904 PMC7888233

[41] Wanat M, Hoste M, Gobat N, et al. Transformation of primary care during the COVID-19 pandemic: experiences of healthcare professionals in eight european countries. Br J Gen Pract. 2021;71(709):e634–e642. 33979303 10.3399/BJGP.2020.1112 PMC8274627

[42] Eisele M, Pohontsch NJ, Scherer M. Strategies in primary care to face the SARS-CoV-2/COVID-19 pandemic: an online survey. Front Med. 2021;8:613537–613537. 10.3389/fmed.2021.613537 PMC8206267 34150788

[43] Mintzberg H. Mintzberg on management: inside our strange world of organizations. New York: Simon and Schuster; 1989.

[44] Yukl G. Leadership in organizations. 7th ed. Upper Saddle River (NJ): Pearson; 2010.

[45] Mintzberg H. Managing the myths of health care. World Hosp Health Serv. 2012;48(3):4–7. 23342753

[46] Abbott A. The system of professions: an essay on the division of expert labor. Chicago (IL): university of Chicago Press; 1988.

[47] Freidson E, editor. Professional dominance: the social structure of medical care. Boca Raton (FL): Routledge; 1971.

[48] Høye S, Braend AM, Spehar I. Quality improvement and antimicrobial stewardship in general practice – the role of the municipality chief medical officer. A qualitative study. Scand J Prim Health Care. 2020;8(3):352–359. 10.1080/02813432.2020.1794400 PMC7470114 32735152

